# Functions, disabilities and perceived health in the first year after total knee arthroplasty; a prospective cohort study

**DOI:** 10.1186/s12891-018-2159-7

**Published:** 2018-07-25

**Authors:** Danielle D. P. Berghmans, Antoine F. Lenssen, Pieter J. Emans, Rob A. de Bie

**Affiliations:** 1 Department of Physical therapy, Maastricht University Medical Center +, PO 5800, 6202 AZ Maastricht, The Netherlands; 2 Department of Orthopedics, Maastricht University Medical Center +, PO 5800, 6202 AZ Maastricht, The Netherlands; 3 Department of Epidemiology, Maastricht University, P.O. Box 616, 6200 MD Maastricht, The Netherlands; 4 Maastricht University/CAPHRI School for Public Health and Primary Care, P.O. Box 616, 6200 MD Maastricht, The Netherlands

**Keywords:** Osteoarthritis, TKA, Total knee arthroplasty

## Abstract

**Background:**

In end-stage knee osteoarthritis total knee arthroplasty (TKA) is an effective intervention to reduce pain and improve functioning in the majority of patients. However, after TKA some patients still experience pain, loss of function, deficient muscle strength or reduced walking speed. This study systematically assesses patients’ functions, disabilities and health before TKA and at short- (3 months) and long-term (12 months) on all International Classification of Functioning, Disability and Health domains.

**Methods:**

In this prospective cohort study 150 patients underwent the following tests before and at 3 and 12 months after surgery: Western Ontario and McMaster Universities Arthritis Index, Short Form 12, Knee Society Score, Patient Specific Functioning Scale, knee range of motion, quadriceps and hamstring strength, gait parameters, global perceived effect (only after surgery). All data was analyzed with repeated measures ANOVA for all measurement time points.

**Results:**

Despite increased gait speed, quadriceps strength and scores on questionnaires being above pre surgical levels, patients do not reach levels of healthy persons. Walking speeds approach normal values and are higher in our study compared with the literature. Quadriceps strength stays at around 70 till 80% of norm values. However, dissatisfaction rates are below 10%, which is low compared to the literature.

**Conclusions:**

Quality of life, activities, muscle strength and gait parameters improve significantly after TKA. However, some complaints regarding activities and walking speed remain. Most striking outcome is the remaining deficit in quadriceps strength.

## Background

Osteoarthritis (OA) is one of the ten most disabling diseases in developed countries: 9.6% of all men and 18.0% of all women over 60 years of age have symptomatic osteoarthritis [1]. While pain is the most prominent symptom, 80% of these patients have limitations in movement, and 25% cannot perform daily activities [1]. An increase in prevalence is expected due to ageing and obesity [1–5].

In end-stage osteoarthritis, joint replacement is an effective intervention to reduce pain and improve functioning in the majority of patients [3, 6–9]. However, it has been reported that 15–30% of patients still experience pain and loss of function after total knee arthroplasty (TKA) [7, 9–12].

Several studies have investigated recovery after TKA. Although most describe a 10–20% improvement in quadriceps strength and gait parameters in comparison to the pre-operative status, values remained lower than in healthy peers or the uninvolved leg [11–19]. A correlation between quadriceps strength and functional performance after TKA [18, 19] seems logical, for functional performance can be assumed to improve with quadriceps strength. Several studies found improvements in patient-reported outcome measures (PROMs), functional status and quality of life. [12–14, 20, 21]. However, patients often did not regain optimal health [22]. No relation with strength is investigated in these studies.

Although several studies investigated aspects of recovery after TKA, no study yet has incorporated all domains of the International Classification of Functioning, Disability and Health (ICF) in their assessment. Interesting findings in the ICF domains may be missed by the limited follow-up, population size and incompleteness of ICF domains of other studies. We therefore performed a prospective cohort study in which 150 patients scheduled for TKA were followed till 1 year after surgery, and in which we systematically assessed all domains of the ICF. Our first objective was to provide a more complete overview of current physical recovery rates of patients with TKA in the first year after surgery in the Netherlands. We hypothesize that the patients in our study will improve on all parameters in the first year after surgery. Our second objective is to generate normative quadriceps and hamstrings strength values for patients receiving a TKA in the Netherlands.

## Methods

### Study design

This prospective cohort study assessed all patients with the same set of measurement instruments during personal follow-up consultations at three time points before and after surgery. We chose as an endpoint a follow-up time of 1 year, as no further major improvement can be expected after this time period [7, 23].

### Patients

Between March 1, 2011 and March 1, 2013, all consecutive patients with knee osteoarthritis scheduled for a TKA at the osteoarthritis clinic of Maastricht University Medical Centre (MUMC+) were informed about the study in writing and verbally at least 1 week before the planned surgery. On the day before surgery, when patients arrived at the hospital, they were contacted by the researcher, and written informed consent was obtained.

At the start of the study, we performed a sample size calculation. Based on the number of determinants and the pragmatic rule of thumb to include ten cases for each determinant we would need at least 120 cases to obtain adequate power (10 × 12 determinants = 120). Since we expected a lost of follow up of 15% we would need 140 subjects. To be on the safe side we included 10 patients more (*N* = 150).

Inclusion criteria were: Dutch speaking patients between 18 and 80 years at the time of surgery, diagnosed with knee osteoarthritis for which primary TKA was indicated. Patients were excluded if they underwent a unicondylar knee arthroplasty (UKA), had a neurological problem influencing ambulation or had an immobile hip or ankle arthrodesis. Severe comorbidities were excluded since all patients had to be eligible for surgery.

The local medical ethics committee of the MUMC+ reviewed and approved the study (NL33015.068.10 / METC 10–2-083). The rights of the subjects were protected under the Helsinki Declaration.

### Surgery

All patients received a cemented Scorpio or Scorpio NRG TKA (Stryker, Kalamazoo, Michigan, USA). After performing a medial parapatellar approach a bony referenced, tibia first technique was used. A tourniquet was only used during the cementation period of the prosthesis. A previous study reported no differences in Range of Motion (ROM), function or Quality of Life between these to prosthesis [24].

### Procedure

After signing informed consent, patients were enrolled in the cohort study. All assessments were performed by the research team the day before surgery and 3 and 12 months after surgery. We chose these time points because of scheduled appointments, enabling us to have personal contact with the patients without involving extra travel time. The patients were not shown the answers or the values obtained in previous sessions.

### Measurements

In addition to the demographic patient characteristics (age, sex, height and weight), the following questionnaires and measurements were performed by a research team using a standardized protocol.

#### Health status questionnaires

The *Western Ontario and McMaster Universities Osteoarthritis Index (WOMAC)* is a self-administered disease-specific health questionnaire designed to measure the functional ability of the osteoarthritic hip and knee [25]. The WOMAC provides aggregate scores for each of three subscales: joint pain, joint stiffness and function. The WOMAC is a responsive instrument that yields reliable and valid measurements in patients with hip and knee osteoarthritis and has been extensively used to evaluate this patient population [9, 25–27]. The 5-point Likert version of this measure was used in our study. The scale was transformed to a range from 0 to 100 points (100 being the best score).

The *Patient Specific Functional Scale (PSFS)* records patients’ perceptions of their disabilities [28]. Patients define their main complaints (i.e. difficulties performing certain activities) and rate the difficulty of performance on an 11-point numerical rating scale (NRS) (10 = no problems; 0 = impossible) [28] The three main complaints had to be defined as specifically as possible, and had to cause difficulties related to the osteoarthritis of the knee. The PSFS is a reliable and responsive measure in this population, [29, 30] and its validity has been confirmed in a population of patients with knee problems [29].

The *Knee Society Score (KSS)* is a knee-joint specific questionnaire and consists of two parts: a knee score (0–100, 100 being the best score) and a function score (0–100, 100 being the best score) [31]. The KSS is a valid and responsive measure in a population of patients after TKA [32].

The *Short Form 12 (SF12)* is a generic multidimensional questionnaire measuring quality of life from a patient’s perspective. It is a short version of the SF36 and includes two components (physical and mental health), representing these respective domains (scale 0–100,100 being the best score). It is a valid, reliable and responsive measure in a general population and easy to administer [33].

The *global perceived effect (GPE)* is a 2-item scale on which patients can rate their overall recovery since a predefined point (in this study pre-surgical function) in time and their satisfaction with the treatment, on 7-point Likert scales (ranging from 2 [satisfied] to 14 [dissatisfied]). Its reliability and validity are good in patients with musculoskeletal disorders [34].

#### Physical performance tests

*Muscle strength was* assessed with a Biodex® System 3 Pro dynamometer (Biodex System 3 Pro Dynamometer, Biodex Medical Systems, Inc., Biodex Medical Systems, USA). Isokinetic strength (60^0^/s and 180^0^/s, in Nm) of the quadriceps and the hamstrings was measured using respectively five and ten repetitions. The peak volitional values were used in the analysis. The Biodex® is a reliable and valid isokinetic dynamometer [35].

*ROM* was measured with a long-arm goniometer, (Goniometer, Long Arm, Gymna, Belgium), according to Lenssen et al. [36] Extension and flexion were measured in supine position, with hyperextension noted as a positive value. Measuring ROM with a long-arm goniometer has been reported to be valid and reliable at group level [36].

The *gait parameters* of step length and walking speed were measured with the GAITRite® system (CIR systems, PA, USA), a highly valid and reliable tool to assess temporospatial gait parameters in patients undergoing a TKA [37].

### Statistical analyses

Analyses were performed with SPSS for Windows version 23. [38] Means and standard deviations were calculated to describe characteristics. Repeated Measures ANOVA were performed to test for significant differences between baseline till 3 months after surgery and between 3 and 12 months after. A significance level of *p* < .05 was used. A Bonferroni correction was used to correct for multiple testing.

Patients who dropped out during the test period were not replaced. All available data was analyzed; in case data was missing, the mean value of the parameter at that time point was imputed.

We also compared the strength of the quadriceps and hamstrings measured in our population with that of controls. We built a norm data set, consisting of 245 patients, 166 women (mean age 58.4 years [10.1], weight 69.6 kg [10.9]) and 129 men (56.2 years [10.7], weight 83.9 kg [10.6]). We tested their isokinetic quadriceps and hamstrings strength at an angular speeds of 60°/s and 180°/s. We calculated formulas (Table 1) for both sexes in both angular speeds for the quadriceps and hamstrings. These formulas were used to calculate the mean norm values for our population. These values and the percentage of the norm values are presented.

**Table 1:** Isokinetic strenght formula

		Men	Women
Quadriceps	*60°/s*	305–2,67×age	172–1,42×age
	*180°/s*	207–1,96×age	108–0,94×age
Hamstrings	*60°/s*	193–1,66×age	116–1,01×age
	*180°/s*	149–1,36×age	89–0,81×age

Table 1 shows the fomula for calculating the isokinetic strength for a healthy population. Age in years

## Results

Between March 1, 2011 and March 1, 2013, we included 150 patients, 71 men and 79 women, with a mean age of 64.7 ± 8.0 years. The majority of patients had surgery of the right knee (right 89, left 61) and mean BMI was 31.2 kg/m^2^ for men and 30.7 kg/m^2^ for women (Table 2). We lost 4 patients at 3-months follow-up and an additional 3 at the 12-month measurement. Not all patients were able to come to the hospital for the follow-up measurements, so data on physical tests (strength, temporospatial gait parameters and ROM) were unavailable for 7 patients at 3-months and for 9 patients at the 12-month measurement (4 of whom were the same as those at the three-month measurement).

**Table 2:** Patient Characteristics

	Men	Women
Age (y) (sd)	64.2 (8.8)	65.1 (7.4)
Height (m) (sd)	1.75 (0.06)	1.66 (0.06)
Weight (Kg) (sd)	95.7 (13.6)	84.6 (15.5)
Body Mass Index (Kg/m^2^) (sd)	31.2 (4.2)	30.7 (5.5)

Age, height, weight and body mass index for men and women. *sd* standard deviation

With the exception of the SF12, patients improved significantly on all questionnaires over 3 and 12 months. The largest improvement occurred within the first 3 months. The largest and significant improvement of the SF12 Physical component was only between baseline and 3 months (Table 3)*.*

**Table 3:** Questionnaires

	Baseline	3 months		12 months	
			*sig.*		*sig.*
Short Form - 12; Physical (sd)	33.5 (7.9)	38.8 (7.7)	*0.000*	39.3 (9.2)	*1.000*
Short Form - 12; Mental (sd)	44.7 (10.6)	45.0 (9.6)	*1.000*	46.4 (9.2)	*0.164*
WOMAC Pain (sd)	10.6 (4.1)	16.0 (4.1)	*0.000*	17.6 (4.4)	*0.000*
WOMAC Stifness (sd)	4.1 (1.9)	4.9 (1.9)	*0.000*	5.8 (1.9)	*0.000*
WOMAC Function (sd)	39.0 (12.3)	54.4 (13.0)	*0.000*	58.2 (12.9)	*0.000*
WOMAC Total (sd)	54.1 (16.2)	75.4 (17.6)	*0.000*	81.7 (18.0)	*0.000*
PSFS 1 (sd)	1.9 (2.0)	2.8 (3.4)	*0.000*	6.6 (3.7)	*0.000*
PSFS 2 (sd)	2.2 (2.1)	4.8 (3.6)	*0.000*	6.8 (3.4)	*0.000*
PSFS 3 (sd)	2.4 (2.4)	4.9 (3.6)	*0.000*	6.9 (3.4)	*0.000*
Knee Society Score; Knee (sd)	52.5 (16.4)	76.1 (17.3)	*0.000*	84.1 (17.5)	*0.000*
Knee Society Score; Function (sd)	57.2 (13.1)	69.4 (15.4)	*0.000*	74.8 (18.5)	*0.000*

*N* number, *sd* standard deviation, *WOMAC* Western Ontario and McMaster Universities Osteoarthritis Index, *PSFS* Patient Specific Functional Complaint, *Sig p*-value

The scores on the questionnaires are given at baseline and 3 and 12 months after surgery. Significance of progression from baseline to 3 months and from 3 till 12 months

**Fig. 1:**
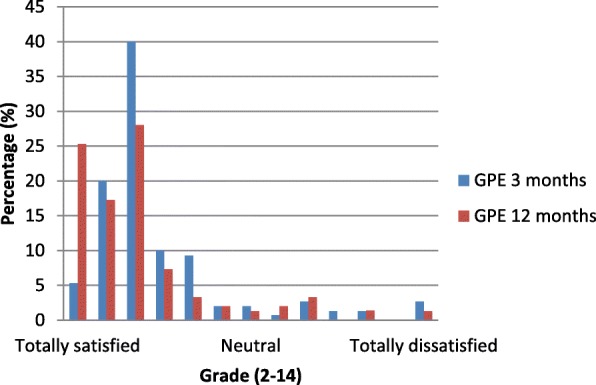
shows the Global Perceived Effect scores 3 and 12 months after TKA, with percentages. GPE; Global Perceived Effect

The GPE is shown in Fig. 1*.* After 3 months, 5.3% of all patients reported to have fully recovered from surgery, and after 1 year 25.3% did so. After 3 months, 2.7% of all patients were totally dissatisfied with the result and the treatment, against 1.3% after 1 year. Overall, the majority of patients were satisfied with the result, but had some residual complaints.

**Fig. 2:**
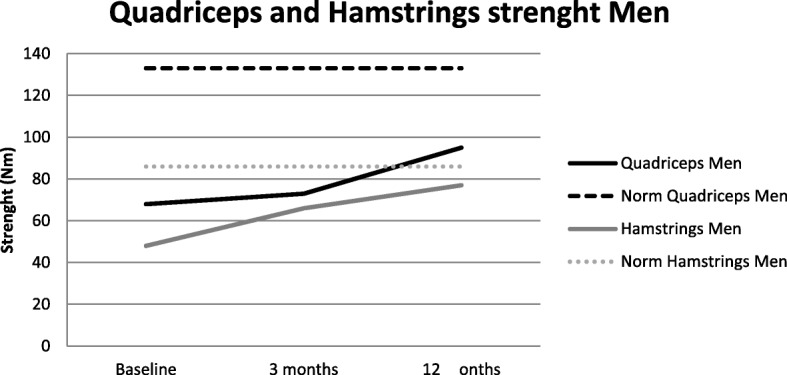
Strength men. Present the development of the Isokinetic Quadriceps and Hamstrings strength in the first year after a TKA in men and the reference values for this population

**Fig. 3:**
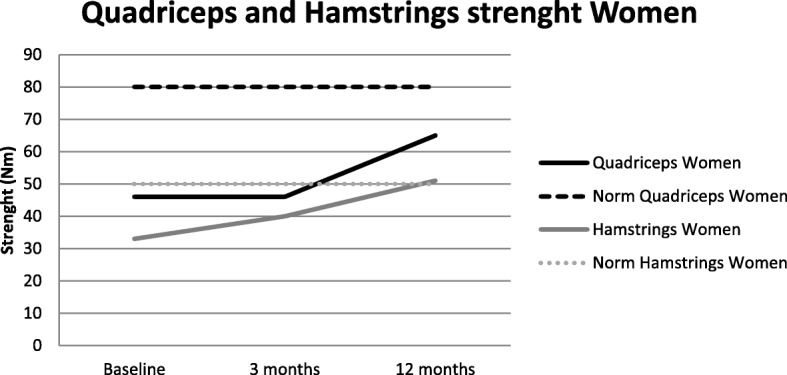
Strength women. Present the development of the Isokinetic Quadriceps and Hamstrings strength in the first year after a TKA in women and the reference values for this population

Figure 2 and 3 show respectively the 60°/s isokinetic quadriceps and hamstrings muscle strength in the peri-operative phase of men and women. After 3 months the quadriceps isokinetic strength measured at 60°/s speed is back on pre-surgical level for women (baseline 43.4 Nm [27.8]; 3 months 46.1 Nm [17.1]; *significance 1.000*) and for men (baseline 67.9 Nm [35.5]; 3 months 73.3 Nm [26.2]; *significance 0.521*). The quadriceps 180°/s only improved significantly in men in 3 3 months *(*baseline 43.3 Nm [23.0]; 3 months 50.5 Nm [17.4]; *significance 0.005)*, women were back on pre-surgical level (baseline 29.2 Nm [16.4]; 3 months 30.0 Nm [11.7]; *significance 1.000*). Over 12 months a significant improvement was demonstrated, at both angular speeds and for both sexes (women 60°/s: 64.5 Nm [18.9] 180°/s: 38.2 [11.8]; men 60°/s: 95.2 Nm [30.5] 180°/s: 61.8 [19.1]). 60 °/s Isokinetic Hamstrings strength in men and women improved significantly in the first year after surgery, compared to pre-surgical values (women; baseline 33.3 Nm [18.9]; 3 months 39.8 Nm [16.7]; *significance 0.009;* 12 months 50.7 Nm [16.2]; *significance 0.000;* men; baseline 48.0 Nm [23.4]; 3 months 65.6 Nm [22.8]; *significance 0.000;* 12 months 77.2 Nm [26.1]; *significance 0.000*). 180°/s Isokinetic Hamstrings strength only improved significant in in men in both time periods (baseline 37.8 Nm [18.6]; 3 months 47.1 Nm [17.8]; *significance 0.000;* 12 months 54.4 Nm [20.9]; *significance 0.002.* and in women only between 3 and 12 months (baseline 24.4 Nm [12.6]; 3 months 27.1 Nm [11.3]; *significance 0.263;* 12 months 33.3 Nm [11.6]; *significance 0.000.)*

We also compared muscle strength of our patients with that of healthy individuals. At baseline, our patients had 51.6 to 63.3% of the quadriceps strength of healthy persons, and 56.8–69.5% of the hamstrings strength. At 3 months, values were 55.7 to 64.4% of the quadriceps and 76.1–79.5% of the hamstrings, respectively, and at 12 months 72.0 to 81.7% and 88.4 to 100.7%, respectively.

Table 4 lists the ROM values. The mean baseline flexion was 120°. Flexion was limited immediately after surgery, but increased again during the first weeks. After three months, flexion had returned almost to baseline value, although there was still a significant difference. At 12 months, the flexion had returned to baseline level. Overall, extension ROM did not change significantly after a TKA.

**Table 4:** ROM

	Baseline	3 months		12 months	
			*sig.*		*sig.*
Flexion (°) (sd)	120.1 (12.6)	114.9 (13.7)	*0.001*	120.6 (14.7)	*0.000*
Extension (°) (sd)	−2.3 (5.7)	−2.5 (5.3)	*1.000*	−1.3 (5.7)	*0.085*

*n* number, *Sig. p* -value

Flexion and extension values prior to surgery and 3 and 12 months after surgery, in degrees. Significance of progression from baseline to 3 months and from 3 till 12 months

Table 5 shows walking speed and step length. The step length of the healthy and surgical leg increased significantly over time. With increasing step length, walking speed also increased significantly over time after TKA (98.9 cm/s at baseline, 108.4 cm/s at 3 months and 117.0 cm/s at 12 months). Improvement of walking speed between baseline and the 3-month measurement was comparable to that between the 3- and 12-month measurements. The largest improvement in step length was between baseline and 3-month measurements.

**Table 5:** Walking speed and step length

		Baseline	3 months		12 months	
				*sig.*		*sig.*
Walking speed (cm/s) (sd)		98.9 (23.3)	108.4 (19.9)	*0.000*	117.0 (19.9)	*0.000*
Step lenght	Surgical leg (cm) (sd)	57.6 (11.2)	61.9 (8.3)	*0.000*	64.1 (8.5)	*0.000*
	Healthy leg (cm) (sd)	58.0 (9.9)	62.2 (8.6)	*0.000*	64.8 (8.7)	*0.000*

*n* number, *Sig. p*- value

Walking speed and step length pre-surgical and 3 and 12 months after TKA with the significance level

## Discussion

This study reports on the first year of physical recovery of patients undergoing a TKA in the Netherlands and could therefore be useful in informing patients about prognostic consequences of a TKA.

The most remarkable finding in this study is the persistent limited muscle strength. Whilst other outcome measures improved to nearly normal values compared to the healthy population, quadriceps strength lagged behind. Before surgery, strength was half of that of matched healthy persons, a higher deficit than reported in the literature [12, 15]. Three months after surgery, mean strength was comparable to pre-surgical values. Although strength increased from 3 till 12 months after TKA, it never reached ‘healthy’ values. We do not expect that pain has a role in the muscle strength deficit since a large amount of patient did not experienced pain during strength measurement after the surgery, in contrast with the measurement pre-surgically. One reason could be the changed kinematics of the knee (altered patellofemoral kinematics) and/or muscle loss due to surgery. A change in strength was seen in an in vitro study by Ostermeier et al., who compared hinged and non-hinged TKAs [39]. Further, during therapy focus is on functional training and isokinetic strength measurement is not performed standardly. Our large study population underlined the muscle deficit already reported in other studies with a smaller sample size [12, 15–19]. In these studies even a higher deficit was described 3 months after surgery [12, 18]. Further, the total gain in muscle strength in the first year was higher in our study compared to the literature [15]. This could be due to the larger improvement in the first months compared with the literature. A reason could be the health care system in the Netherlands. The main part of all patients receive routinely a prescription for physical therapy after the TKA surgery for 1 year physical therapy. Generally, they have therapy twice a week. This could result in a faster and larger improvement of muscle strength which focuses on activity level, like walking with and without crutches and walking stairs. This is in contrast with the amount of physical therapy patients receive in other countries worldwide. Bade et al. described only that 26% of all patients receive outpatient physical therapy [12]. This could also possibly explain the smaller deficit of walking speed in our study. Walking speed in our pre-surgical [12, 13] and post-surgical patients is higher than reported in the literature in which patients with a TKA walked 20% more slowly than healthy individuals 1 year after surgery [8, 13, 17]. Before surgery, the walking speed of patients in our study (98,9 cm/s) was 20.0–26.1% lower than in a healthy population (healthy walking speed for women [60–69 years] 1.24 m/s and for men [60–69 years] 1.34 m/s) [40]. After surgery, walking speed (108.4 cm/s) increased but remained lower than in healthy persons (between 81.0–87.3% of the healthy walking speed) [40]. After 12 months, walking speed (117.0 cm/s) still increased, but did not reach the healthy level (attaining between 87.3 and 94.2% of the walking speed of healthy individuals). However, in terms of managing pedestrian street crossings with lights (which are designed for 1.2 m/s), 81% of our population were unable to cross the street safely before their surgery. After 3 months, this percentage had decreased to 65%, but after 12 months it was still 49%.

Pua et al. [41] investigated the relationship between walking speed and physical parameters in the first 16 weeks after TKA. They found ipsilateral quadriceps strength as strongest predictor for walking speed. No linear relationship existed till 111 N, a steep rise in gait speed was observed with every gain in muscle strength. After 111 Newton the speed increased more gradually [41]. However, according to Alnahdi et al. [42], the influence of quadriceps strength on gait patterns is only moderate till 6 months and in the period till 1 year it even decreases.

All questionnaires regarding level of functioning, quality of life and patient-specific complaints yielded lower scores compared to healthy controls, both pre- and postsurgical. (For healthy peers, we assumed highest possible score on the WOMAC, KSS and PSFS [related to knee problems] may be expected. For the SF12 we used reference data from the United states of America in which the healthy population had a score on the mental part of 51.6 and 43.9 on the physical part [43]). However, a large and significant increase was reported on all questionnaires over the 12 months following surgery. This is in agreement with previous studies [4, 9, 12–15, 18, 20–22]. Only, our population seemed to recover faster; they performed on pre-surgical level at 3months, while in other studies this took about 6 months [12]. Again, this could be due to the amount of physical therapy in the Netherlands, focused on activities like walking, walking stairs and making transfers.

Possible due to the faster increase in strength, walking speed and questionnaires, our patients were more satisfied compared to those in other studies, as seen on the GPE (8.7% dissatisfied at 3 months, 7.9% dissatisfied at 12 months, whereas in other studies 15–30% of the patients were dissatisfied) [7, 9–12]. However, a relation between satisfaction and improvement in pain, function and handicap is difficult, and therefore, according to Genet et al. satisfaction should be investigated as an independent parameter [14].

Nonetheless, patients’ physical capability can be satisfying, their spare capacity could be less, giving a higher risk for frailty in case of a trauma or hospitalization. Therefore, despite of the importance of their satisfaction as success indicator for surgery, measuring their functional activity level is an important indicator.

### Limitations

We decided to use performance tests to assess functions and questionnaires to measure activities, so the results regarding activities are from the patients’ view and could therefore be subjective.

Another limitation is that we do not have information about osteoarthritis in other joints of the patients. Which might have an influence on the functional performance.

Our findings confirm that a TKA improves quality of life for patients with knee osteoarthritis and can be used to inform patients about possible prognostic consequences of a TKA, which is imported in patient-centered care. However, patients do not reach the values attained by healthy persons, and complaints persist in the first year after surgery. Quadriceps strength in particular remains limited, which may be a reason for persistent complaints. In our opinion most muscle deficits are not noticed during daily activities. However, in case of illness of (surgical) stress patients have less spare capacity and will have a higher chance to become frail. Physical therapy focuses on ROM and daily activities, but testing and training quadriceps strength until normal values are attained (if possible) is an important part of the therapy. As mentioned earlier, further research should focus on the effect of more progressive resistance training on the remaining muscle strength deficits in the first year after TKA. Besides this, further investigation in pathophysiology of muscle weakness is necessary.

## Conclusions

Quality of life, activities, muscle strength and gait parameters improve significantly after a TKA. However, complaints on activities and walking speed remain. Most striking was the limited quadriceps strength, which we believe may restrict patients in daily life. Therefore, future studies should address the impact of strength training after a TKA on the improvement in muscle strength and daily activities.

## Abbreviations

GPE: Global Perceived Effect
ICF: International Classification of Functioning, Disability and Health
KSS: Knee Society Score
MUMC: Maastricht University Medical Centre
OA: Osteoarthritis
PROM: Patient-Reported Outcome Measures
PSFS: Patient Specific Functional Scale
ROM: Range of Motion
SF12: Short Form 12
TKA: Total Knee Arthroplasty
UKA: Unicondylar Knee Arthroplasty
WOMAC: Western Ontario and McMaster Universities Osteoarthritis Index
